# Inflammatory Markers and Severity in COVID-19 Patients with Clostridioides Difficile Co-Infection: A Retrospective Analysis Including Subgroups with Diabetes, Cancer, and Elderly

**DOI:** 10.3390/biomedicines13010227

**Published:** 2025-01-17

**Authors:** Teodor Cerbulescu, Flavia Ignuta, Uma Shailendri Rayudu, Maliha Afra, Ovidiu Rosca, Adrian Vlad, Stana Loredana

**Affiliations:** 1 Department III—Microscopic Morphology, Discipline of Cellular and Molecular Biology, “Victor Babes” University of Medicine and Pharmacy, Eftimie Murgu Square 2, 300041 Timisoara, Romania; teodor.cerbulescu@umft.ro; 2 Doctoral School, “Victor Babes” University of Medicine and Pharmacy Timisoara, Eftimie Murgu Square 2, 300041 Timisoara, Romania; 3 Department of Infectious Disease, “Victor Babes” University of Medicine and Pharmacy Timisoara, Eftimie Murgu Square 2, 300041 Timisoara, Romania; ovidiu.rosca@umft.ro; 4 Centre for Molecular Research in Nephrology and Vascular Disease, Faculty of Medicine, “Victor Babes” University of Medicine and Pharmacy Timisoara, Eftimie Murgu Square 2, 300041 Timisoara, Romania; vlad.adrian@umft.ro; 5 Medical School, Gitam Institute of Medical Sciences and Research, Visakhapatnam 530045, India; uma.shailendri@gmail.com; 6 Medical School, Deccan College of Medical Sciences, Hyderabad 500058, India; malihaafra04@gmail.com; 7 Department of Internal Medicine II, Division of Diabetes, Nutrition and Metabolic Diseases, “Victor Babes” University of Medicine and Pharmacy Timisoara, Eftimie Murgu Square 2, 300041 Timisoara, Romania; 8 Department I, Discipline of Anatomy and Embryology, “Victor Babes” University of Medicine and Pharmacy, Eftimie Murgu Square 2, 300041 Timisoara, Romania; loredana.stana@umft.ro

**Keywords:** COVID-19, *Clostridioides difficile* infection, inflammatory markers, severe outcomes

## Abstract

Background and Objectives: The interplay of Severe Acute Respiratory Syndrome Coronavirus 2 (SARS-CoV-2) infection and *Clostridioides difficile* infection (CDI) poses a critical clinical challenge. The resultant inflammatory milieu and its impact on outcomes remain incompletely understood, especially among vulnerable subgroups such as elderly patients, those with diabetes, and individuals with cancer. This study aimed to characterize inflammatory markers and composite inflammatory severity scores—such as Acute Physiology and Chronic Health Evaluation II (APACHE II), Confusion, Urea, Respiratory rate, Blood pressure, and age ≥ 65 years (CURB-65), National Early Warning Score (NEWS), and the Systemic Immune-Inflammation Index (SII)—in hospitalized Coronavirus Disease 2019 (COVID-19) patients with and without CDI, and to evaluate their prognostic implications across key clinical subgroups. Methods: We conducted a retrospective, single-center study of 240 hospitalized adults with Reverse Transcription Polymerase Chain Reaction (RT-PCR)-confirmed COVID-19 between February 2021 and March 2023. Of these, 98 had concurrent CDI. We collected baseline demographics, comorbidities, and laboratory parameters including C-reactive protein (CRP), Interleukin-6 (IL-6), ferritin, neutrophil and lymphocyte counts, albumin, platelet counts, and calculated indices (C-reactive protein to Albumin Ratio (CAR), Neutrophil-to-Lymphocyte Ratio (NLR), Prognostic Nutritional Index (PNI), SII). Patients were stratified by CDI status and analyzed for inflammatory marker distributions, severity scores (APACHE II, CURB-65, NEWS), and outcomes (Intensive Care Unit (ICU) admission, mechanical ventilation, mortality). Subgroup analyses included diabetes, elderly (≥65 years), and cancer patients. Statistical comparisons employed *t*-tests, chi-square tests, and logistic regression models. Results: Patients with CDI demonstrated significantly higher CRP, IL-6, SII, and CAR, coupled with lower albumin and PNI (*p* < 0.05). They also had elevated APACHE II, CURB-65, and NEWS scores. CDI-positive patients experienced increased ICU admission (38.8% vs. 20.5%), mechanical ventilation (24.5% vs. 12.9%), and mortality (22.4% vs. 10.6%, all *p* < 0.05). Subgroup analyses revealed more pronounced inflammatory derangements and worse outcomes in elderly, diabetic, and cancer patients with CDI. Conclusions: Concurrent CDI intensifies systemic inflammation and adverse clinical trajectories in hospitalized COVID-19 patients. Elevations in inflammatory markers and severity scores predict worse outcomes, especially in high-risk subgroups. Early recognition and targeted interventions, including infection control and supportive measures, may attenuate disease severity and improve patient survival.

## 1. Introduction

The global burden of COVID-19 infections, driven by the SARS-CoV-2 virus, continues to pose formidable challenges to healthcare systems worldwide, despite the end of the pandemic [1,2]. Vaccination efforts and antiviral therapies could not prevent the infection of approximately 700 hundred million people and the death of almost 7 million people [3,4]. Still, the hospitalizations for COVID-19-related pneumonia and acute respiratory distress syndrome remain substantial [5,6]. As the pandemic evolves, it has become evident that co-infections can exacerbate the disease course in susceptible patients, heightening morbidity and mortality [7]. Among the most concerning of these co-infections is *Clostridioides difficile* infection (CDI), a common and serious nosocomial infection associated with antibiotic exposure and healthcare contact [8]. CDI typically manifests as colitis and severe diarrhea but can have systemic implications [9]. In the context of COVID-19, patients may be predisposed to CDI through frequent antibiotic use, altered gut microbiota, and immune dysregulation [10,11,12].

The interplay between COVID-19 and CDI is not limited to gastrointestinal symptoms. Emerging evidence suggests that co-infection may amplify systemic inflammation, potentially leading to a more severe respiratory and hemodynamic compromise [13,14,15]. Understanding the inflammatory milieu is crucial, as inflammatory biomarkers and severity scores often guide clinical decisions.

Traditional inflammatory markers, including C-reactive protein (CRP), Interleukin-6 (IL-6), ferritin, and composite indices such as the Neutrophil-to-Lymphocyte Ratio (NLR) and the Prognostic Nutritional Index (PNI), have been studied extensively in COVID-19 [16,17,18,19]. Additionally, severity scores like APACHE II, CURB-65, and NEWS have proven utility in predicting outcomes in critical illness [20,21,22]. The Systemic Immune-Inflammation Index (SII), based on platelet, neutrophil, and lymphocyte counts, offers a novel window into the host response [23].

However, the additive or synergistic effect of CDI on these inflammatory markers and scores in COVID-19 remains underexplored. Moreover, populations with aging, diabetes, and cancer patients are believed to be particularly vulnerable to severe outcomes from COVID-19 and CDI due to specific immunosuppressive and inflammatory mechanisms. Aging leads to diminished immune responses, diabetes is linked to chronic inflammation and weakened immunity, and cancer, especially under chemotherapy, disrupts immune defenses [24,25]. These conditions exacerbate responses to infections like SARS-CoV-2 and *Clostridioides difficile*. Therefore, this study aims to comprehensively investigate the relationship between CDI, inflammatory markers, and clinical severity scores in COVID-19 patients. Through detailed subgroup analyses, we seek to identify prognostic factors and potential targets for early intervention to improve clinical management and patient survival in this complex co-infection scenario.

## 2. Materials and Methods

### 2.1. Study Design and Population

This retrospective cohort study was conducted at a tertiary-care academic hospital in Timisoara, Romania (Eastern Europe). We reviewed the medical records of adult patients (≥18 years) admitted with laboratory-confirmed COVID-19, diagnosed via RT-PCR, between February 2021 and March 2023. Among these, we identified patients who had concurrent CDI, defined by a positive stool PCR test for *C. difficile* toxin B and consistent clinical symptoms. We included 240 patients in total: 98 patients constituted the COVID + CDI group and 142 formed the COVID-only group. We excluded patients with known inflammatory bowel disease, chronic immunosuppressive therapy, or incomplete laboratory datasets. The hospital’s institutional review board approved the study protocol, and the requirement for informed consent was waived due to the retrospective design.

### 2.2. Data Collection and Laboratory Measurements

Demographic data, comorbidities (including hypertension, diabetes mellitus, malignancy, and chronic kidney disease), and clinical outcomes were retrieved. Inflammatory markers recorded within 48 h of admission included CRP, IL-6, ferritin, albumin, complete blood counts (for neutrophil, lymphocyte, and platelet counts), and relevant composite indices. The following indices and scores were calculated: NLR = Neutrophils/Lymphocytes; PNI = (10 × Albumin [g/L]) + (0.005 × Lymphocyte count [×10^9^/L]); CAR = CRP (mg/L)/Albumin (g/L); SII = (Platelets × Neutrophils)/Lymphocytes. For severity assessment, APACHE II, CURB-65, and NEWS were computed. Standardized laboratory methods and scoring algorithms were applied to ensure measurement consistency.

Clinical laboratory parameters were measured using standardized and validated assays to ensure accuracy and consistency. CRP levels were quantified through high-sensitivity immunoturbidimetric assays performed on an automated clinical chemistry analyzer. IL-6 concentrations were determined using ELISA kits following the manufacturer’s instructions. Ferritin levels were assessed via chemiluminescent immunoassays on the same analyzer. Albumin was measured using the bromocresol green method, while complete blood counts, including neutrophil, lymphocyte, and platelet counts, were obtained using an automated hematology analyzer. Platelet counts were verified manually when discrepancies were detected. Composite indices such as NLR, PNI, CAR, and SII were calculated based on the obtained laboratory values. All laboratory measurements were conducted within the first 48 h of patient admission to ensure timely and relevant data collection. Quality control procedures were strictly followed, including the use of internal controls and participation in external proficiency testing programs, to maintain the reliability of the laboratory results.

### 2.3. Definitions and Outcomes

COVID-19 severity was defined as per established guidelines, with severe disease indicated by ICU admission or mechanical ventilation. CDI severity was classified using standard clinical criteria, incorporating stool frequency and evidence of systemic involvement [26]. The primary outcomes were ICU admission, mechanical ventilation, and in-hospital mortality. Secondary outcomes included hospital length of stay and severity score correlations with laboratory parameters. Subgroup analyses focused on patients ≥65 years, those with diabetes, and individuals with a documented malignancy, to evaluate variations in inflammatory profiles and outcomes.

In this study, detailed demographic and clinical data were collected to ensure comprehensive subgroup analyses. The types of cancer recorded included solid tumors and hematological malignancies, with specific classifications based on the latest WHO guidelines. For diabetes evaluation, parameters such as Hemoglobin A1c (HbA1c) and fasting glucose were utilized to assess glycemic control. Additionally, diabetes was categorized into type 1 and type 2 based on patient medical history, treatment regimen, and diagnostic criteria from the American Diabetes Association.

### 2.4. Statistical Analysis

Utilizing a power analysis with an expected medium effect size (Cohen’s d = 0.5), a significance level (α) of 0.05, and a desired power of 80%, a minimum of 192 patients (96 per group) was required. To account for potential exclusions and ensure robustness, a total of 240 patients that met the inclusion criteria were retrospectively selected through consecutive sampling from the hospital’s electronic medical records.

Statistical analyses were performed using SPSS version 28. Continuous variables are expressed as mean ± standard deviation, and categorical variables as percentages. Group comparisons employed Student’s *t*-tests, Mann–Whitney U tests, chi-square tests, or Fisher’s exact tests as appropriate. Correlations between inflammatory markers, severity scores, and outcomes were assessed by Pearson or Spearman correlation coefficients. Logistic regression identified independent predictors of severe outcomes. A *p*-value < 0.05 was considered statistically significant.

## 3. Results

### Patient Demographics

In Table 1, the demographic and clinical characteristics of patients with both COVID-19 and CDI are compared to those with COVID-19 alone. The average age of patients in the COVID and CDI group was 68.7 years (±12.9) compared to 66.2 years (±11.7) in the COVID-only group, although this difference was not statistically significant (*p* = 0.16). The proportion of male patients was slightly higher in the COVID and CDI group (57.1%) compared to the COVID-only group (53.5%), but this difference did not reach statistical significance (*p* = 0.55). Body Mass Index (BMI) was marginally higher in the COVID and CDI group (29.1 kg/m^2^ ± 5.1) compared to the COVID-only group (27.8 kg/m^2^ ± 4.9), approaching significance (*p* = 0.07). Similarly, the prevalence of hypertension and diabetes mellitus was higher in the COVID and CDI group (74.5% and 52.0%, respectively) compared to the COVID-only group (63.4% and 40.1%), though these differences were not statistically significant (*p* = 0.07 and *p* = 0.08, respectively).

Clinically, patients with both COVID-19 and CDI experienced more severe outcomes. ICU admissions were significantly higher in the COVID and CDI group (38.8%) compared to the COVID-only group (20.5%) with a *p*-value of 0.002. Mechanical ventilation was required more frequently in the COVID and CDI cohort (24.5%) than in the COVID-only group (12.9%), which was statistically significant (*p* = 0.01). Mortality rates were also significantly elevated in the COVID and CDI group (22.4%) compared to the COVID-only group (10.6%) with a *p*-value of 0.01. Additionally, the length of hospital stay was longer for patients with both infections (16.0 days ± 5.9) compared to those with COVID-19 alone (13.5 days ± 5.1), and this difference was statistically significant (*p* = 0.002). These findings suggest that the presence of CDI in COVID-19 patients is associated with more severe clinical outcomes and longer hospitalization.

Table 2 presents a comparison of inflammatory markers and related indices between patients with both COVID-19 and CDI and those with COVID-19 alone. Patients with both infections exhibited significantly higher levels of CRP (92.6 mg/L ± 26.9 vs. 73.1 mg/L ± 24.4, *p* < 0.001) and Interleukin-6 (IL-6) (65.3 pg/mL ± 18.8 vs. 50.9 pg/mL ± 16.2, *p* < 0.001), indicating a heightened inflammatory response. Ferritin levels were also elevated in the COVID and CDI group (589 µg/L ± 161) compared to the COVID-only group (475 µg/L ± 139), with the difference being statistically significant (*p* = 0.001). The NLR was higher in patients with both infections (7.9 ± 2.7) compared to those with COVID-19 alone (6.1 ± 2.1), and this difference was highly significant (*p* < 0.001).

Conversely, patients with both COVID-19 and CDI had lower albumin levels (29.9 g/L ± 5.3) compared to the COVID-only group (33.6 g/L ± 5.5, *p* < 0.001), suggesting poorer nutritional status or greater disease severity. Platelet counts were reduced in the COVID and CDI group (198 × 10^9^/L ± 55) versus the COVID-only group (225 × 10^9^/L ± 60), with a significant *p*-value of 0.002. The CAR was significantly higher in the dual-infection group (3.10 ± 0.85 vs. 2.17 ± 0.72, *p* < 0.001), while the PNI was lower (33.6 ± 4.6 vs. 38.2 ± 5.1, *p* < 0.001). Additionally, the SII was elevated in patients with both infections (1362 × 10^3^ ± 450) compared to those with COVID-19 alone (1087 × 10^3^ ± 372, *p* < 0.001).

Table 3 compares the severity scores at admission between patients with both COVID-19 and CDI and those with COVID-19 alone. The APACHE II score was significantly higher in the COVID and CDI group (17.1 ± 5.2) compared to the COVID-only group (13.9 ± 4.8), with a *p*-value of less than 0.001. Similarly, the CURB-65 score, which assesses pneumonia severity, was elevated in the dual-infection group (2.6 ± 1.0) compared to the COVID-only group (2.1 ± 0.9), and this difference was statistically significant (*p* = 0.001). The NEWS, which predicts clinical deterioration, was also higher in patients with both infections (8.4 ± 2.5) compared to those with COVID-19 alone (6.9 ± 2.3), with a *p*-value of less than 0.001.

Table 4 presents subgroup analyses focusing on elderly patients (aged ≥ 65 years), those with diabetes mellitus, and patients with cancer. Among the elderly subgroup, 67.3% of patients in the COVID and CDI group were aged 65 or older, compared to 55.6% in the COVID-only group; this difference approached statistical significance (*p* = 0.06). ICU admissions within the elderly subgroup were significantly higher in the COVID and CDI group (42.4%) compared to the COVID-only group (21.5%), with a *p*-value of 0.003. In patients with diabetes mellitus, 52.0% of the COVID and CDI group had diabetes compared to 40.1% of the COVID-only group, although this difference was not statistically significant (*p* = 0.08). However, among diabetic patients, those with both COVID-19 and CDI had a significantly higher rate of ICU admission (45.1%) compared to diabetic patients with COVID-19 alone (24.6%, *p* = 0.01). Regarding cancer, 17.3% of patients in the COVID and CDI group had cancer compared to 10.6% in the COVID-only group, but this difference was not statistically significant (*p* = 0.14). Additionally, mortality among cancer patients was higher in the COVID and CDI group (41.2%) compared to the COVID-only group (20.0%), although this difference did not reach statistical significance (*p* = 0.09).

Table 5 explores the correlations between various inflammatory markers and severity scores with key clinical outcomes, including ICU admission, mechanical ventilation, and mortality. CRP showed a moderate positive correlation with ICU admission (r = 0.42, *p* < 0.001), mechanical ventilation (r = 0.40, *p* < 0.001), and mortality (r = 0.36, *p* < 0.001). Interleukin-6 (IL-6) was similarly correlated with these outcomes, exhibiting correlations of r = 0.45 (*p* < 0.001) for ICU admission, r = 0.41 (*p* < 0.001) for mechanical ventilation, and r = 0.38 (*p* < 0.001) for mortality. The CAR demonstrated strong positive correlations with ICU admission (r = 0.49, *p* < 0.001), mechanical ventilation (r = 0.46, *p* < 0.001), and mortality (r = 0.44, *p* < 0.001).

**Figure 1:**
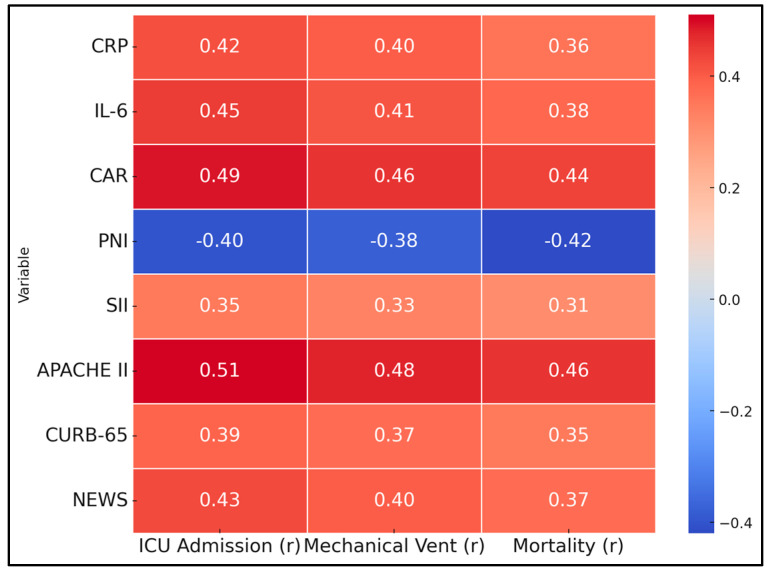
Correlations of inflammatory markers and severity scores with outcomes.

Conversely, the PNI exhibited significant negative correlations with all three outcomes: ICU admission (r = −0.40, *p* < 0.001), mechanical ventilation (r = −0.38, *p* < 0.001), and mortality (r = −0.42, *p* < 0.001). The Systemic Immune-Inflammation Index (SII) was positively correlated with ICU admission (r = 0.35, *p* < 0.001), mechanical ventilation (r = 0.33, *p* = 0.001), and mortality (r = 0.31, *p* = 0.002). Severity scores also showed significant correlations with adverse outcomes: APACHE II (r = 0.51, *p* < 0.001 for ICU admission; r = 0.48, *p* < 0.001 for mechanical ventilation; r = 0.46, *p* < 0.001 for mortality), CURB-65 (r = 0.39, *p* < 0.001; r = 0.37, *p* < 0.001; r = 0.35, *p* < 0.001), and NEWS (r = 0.43, *p* < 0.001; r = 0.40, *p* < 0.001; r = 0.37, *p* < 0.001); all demonstrated significant positive correlations with ICU admission, mechanical ventilation, and mortality (Figure 1).

The presence of both COVID-19 and CDI was a significant predictor, with an odds ratio (OR) of 2.6 (95% CI: 1.50–4.50, *p* < 0.001), indicating that patients with dual infections were 2.6 times more likely to experience severe outcomes compared to those with COVID-19 alone. Interleukin-6 (IL-6) was also a significant predictor, with each 10 pg/mL increase in IL-6 associated with a 1.22-fold increase in the odds of severe outcomes (OR: 1.22, 95% CI: 1.10–1.35, *p* = 0.001). The CAR was another significant predictor, where each 0.5 increase in the CAR was associated with a 1.38-fold increase in the odds of severe outcomes (OR: 1.38, 95% CI: 1.18–1.60, *p* < 0.001).

**Figure 2:**
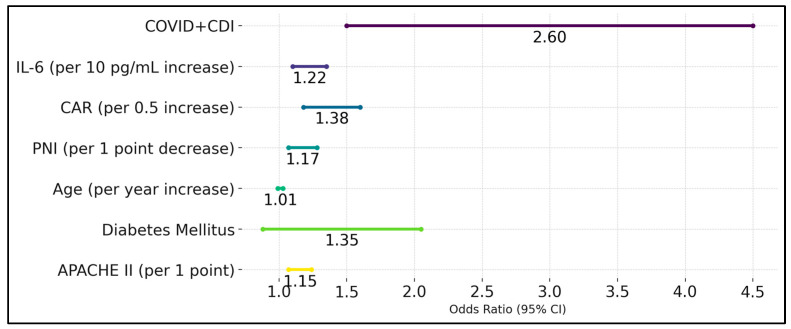
Logistic regression for independent predictors of severe outcomes.

The PNI was inversely related to severe outcomes, with each 1-point decrease in PNI associated with a 1.17-fold increase in the odds of severe outcomes (OR: 1.17, 95% CI: 1.07–1.28, *p* = 0.001). Age was not a significant predictor in the multivariate model (OR: 1.01, 95% CI: 0.99–1.03, *p* = 0.3), nor was diabetes mellitus (OR: 1.35, 95% CI: 0.88–2.05, *p* = 0.15). The APACHE II score remained a significant predictor, with each 1-point increase in APACHE II associated with a 1.15-fold increase in the odds of severe outcomes (OR: 1.15, 95% CI: 1.07–1.24, *p* < 0.001). These findings highlight that dual infection with COVID-19 and *C. diff*, elevated IL-6 and CAR, lower PNI, and higher APACHE II scores are independent predictors of severe clinical outcomes in patients (Table 6 and Figure 2).

Table 7 illustrates the differences in inflammatory markers and severity scores between survivors and non-survivors among patients with CDI. Non-surviving patients exhibited significantly higher levels of CRP (101.2 ± 29.5 mg/L vs. 89.4 ± 25.1 mg/L, *p* = 0.04) and IL-6 (73.3 ± 20.3 pg/mL vs. 63.5 ± 17.9 pg/mL, *p* = 0.02), indicating a more pronounced inflammatory response. Additionally, the CAR was marginally higher in non-survivors (3.33 ± 0.88 vs. 3.02 ± 0.83, *p* = 0.05), and the PNI was significantly lower (31.9 ± 4.3 vs. 34.2 ± 4.5, *p* = 0.01), suggesting poorer nutritional status. Severity was further reflected in higher APACHE II scores among non-survivors (19.5 ± 5.3 vs. 16.4 ± 5.0, *p* = 0.006). While the CURB-65 and NEWS were elevated in non-survivors, these differences did not reach statistical significance.

## 4. Discussion

### 4.1. Analysis of Findings

This study reveals a pronounced interrelationship between CDI co-infection and the inflammatory status of hospitalized COVID-19 patients. Our findings demonstrate that CDI intensifies the underlying immune dysregulation, leading to higher CRP, IL-6, NLR, and SII, as well as worse composite severity scores. Co-infected patients are more likely to require ICU admission and mechanical ventilation, and have a higher risk of death. These observations are consistent with the emerging literature that identifies CDI as a critical complicating factor in respiratory infections, suggesting a bidirectional gut–lung axis where intestinal dysbiosis and systemic inflammation fuel each other.

Subgroup analyses highlight that elderly patients, those with diabetes, and cancer patients are disproportionately affected. These vulnerable groups, already compromised by baseline chronic conditions, appear more susceptible to the additive inflammatory burden imposed by CDI. Interestingly, markers like CAR and PNI proved especially valuable in risk stratification, reflecting the dynamic interplay between inflammation and nutritional status. Severity scores, including APACHE II and NEWS, independently predicted severe outcomes, reinforcing their clinical utility in complex co-infection scenarios.

Our results advocate for heightened clinical vigilance and aggressive preventive strategies for CDI in COVID-19 patients. Early and targeted treatment for CDI, along with careful management of antibiotics, may prevent the escalation of inflammatory cascades. Integration of inflammatory markers and severity indices into routine assessments can guide resource allocation, inform therapeutic choices, and potentially improve outcomes. While our study adds to the growing body of knowledge on co-infections, further prospective research and multi-center validation are needed to refine prognostic models and elucidate the mechanistic pathways linking CDI to worsened COVID-19 outcomes.

In a similar manner, the study by Cojocariu et al. observed a significant correlation between prior COVID-19 infection and the development of *Clostridium difficile* infection, noting particularly that 17.0% of CDI cases had a history of COVID-19 [14]. These patients, primarily elderly and with a history of alcohol consumption, previous hospitalizations, and antibiotic treatments, required higher doses of vancomycin and were more prone to recurrent disease. The study highlighted several risk factors for CDI in COVID-19 patients, including age over 60 years (odds ratio [OR]: 2.591), urban residence (OR: 2.330), and previous antibiotic treatments (OR: 1.909), underscoring the multifaceted nature of CDI risk in this population. Similarly, Patrizia Spigaglia’s review during the COVID-19 pandemic pointed out the overarching impact of the pandemic on hospital-acquired infections, emphasizing that while overall CDI incidence might have decreased due to enhanced infection prevention measures, the critical conditions in hospitals, especially ICUs, and the widespread use of broad-spectrum antibiotics potentially increased the risk and altered the dynamics of CDI [27].

In a similar manner, the study by Markovic-Denic et al. demonstrated that the incidence rates of healthcare-associated *Clostridioides difficile* infections (HA-CDIs) were notably higher in COVID-19 patients, with significant differences in the age of affected patients and the prevalence of antimicrobial therapy prior to infection [28]. Specifically, the incidence of HA-CDIs in COVID-19 patients was found to be three times higher compared to non-COVID-19 patients, with COVID-19 patients frequently receiving two or more antibiotics and other treatments such as proton pump inhibitors and steroids, which are known risk factors for CDI. In contrast, the study conducted by Tossens et al. in Belgium observed a significant decrease in the incidence of CDI during the COVID-19 pandemic, with a reduction of up to 50% compared to the year before the pandemic [29]. This decrease was attributed not to changes in antibiotic use, which remained stable, but rather to the enhanced infection control measures implemented to curb the spread of COVID-19.

In a similar manner, the study by Saied Ali and Sinead McDermott investigated the impact of the COVID-19 pandemic on the incidence of healthcare-associated *Clostridioides difficile* infections in a tertiary healthcare facility in the Republic of Ireland, finding no statistically significant difference in HA-CDI rates between COVID-19 and non-COVID-19 periods, with an incidence rate of 2.1 cases per 10,000 bed-days during the pandemic compared to 1.76 cases in the pre-pandemic period [30]. The antimicrobial consumption and the number of tests performed also did not significantly differ, indicating that the pandemic did not alter the underlying risk factors for HA-CDI in this setting. Similarly, the study conducted by Simona Iftimie et al. in Reus, Spain [31], explored the rates and outcomes of CDI during the COVID-19 pandemic, highlighting a specific increase in CDI infections among oncological patients and those undergoing invasive procedures like colonoscopies or gastroscopies. Despite overall stable rates of CDI, their findings underscored specific subgroups where the incidence increased, possibly due to pandemic-related changes in healthcare practices such as delayed diagnoses and altered treatment protocols.

### 4.2. Study Limitations

Several limitations should be noted. First, this study’s retrospective design introduces inherent biases related to data completeness and patient selection. Although we applied strict inclusion criteria and excluded those with missing critical data, residual confounding cannot be entirely ruled out. Second, the study was conducted at a single tertiary-care center, which may limit the generalizability of the findings to other healthcare settings with different patient populations, antibiotic stewardship policies, or infection control measures. Third, we focused on baseline inflammatory markers without examining longitudinal trends. Serial measurements might offer deeper insights into the temporal dynamics of inflammation and clinical course. Fourth, therapies for COVID-19 and CDI were not standardized, potentially influencing outcomes. Lastly, due to the observational nature of this study, we cannot infer causality but only associations. Future prospective studies and randomized trials are needed to validate these observations and guide evidence-based therapeutic interventions.

## 5. Conclusions

This retrospective cohort study highlights that *Clostridioides difficile* co-infection in hospitalized COVID-19 patients significantly exacerbates systemic inflammation and worsens clinical outcomes. Elevated inflammatory markers such as CRP and IL-6, along with altered immune cell ratios like NLR and SII, were associated with an increased risk of ICU admission, mechanical ventilation, and mortality. Severity scores like APACHE II confirmed that co-infected patients faced more severe disease states. The impact was especially pronounced in elderly, diabetic, and cancer patients, emphasizing the necessity for targeted preventive and therapeutic strategies. Future efforts should focus on early diagnostic stewardship, prompt initiation of appropriate treatments, and comprehensive supportive measures to mitigate the heightened morbidity and mortality associated with this co-infection. Implementing such multifaceted care approaches is crucial for improving patient outcomes in these complex cases.

## Figures and Tables

**Table 1:** Patients’ demographics and outcomes.

Variables	COVID and *C. diff* (n = 98)	COVID-Only (n = 142)	*p*-Value
Age (years)	68.7 ± 12.9	66.2 ± 11.7	0.16
Male (%)	57.1% (56)	53.5% (76)	0.55
BMI (kg/m^2^)	29.1 ± 5.1	27.8 ± 4.9	0.07
Hypertension (%)	74.5% (73)	63.4% (90)	0.07
Diabetes Mellitus (%)	52.0% (51)	40.1% (57)	0.08
Cancer (%)	17.3% (17)	10.6% (15)	0.14
ICU Admission (%)	38.8% (38)	20.5% (29)	0.002
Mechanical Vent (%)	24.5% (24)	12.9% (18)	0.01
Mortality (%)	22.4% (22)	10.6% (15)	0.01
Length of Stay (days)	16.0 ± 5.9	13.5 ± 5.1	0.002

BMI—Body Mass Index; ICU—Intensive Care Unit.

**Table 2:** Inflammatory markers and indices.

Variables	COVID and *C. diff* (n = 98)	COVID-Only (n = 142)	*p*-Value
CRP (mg/L)	92.6 ± 26.9	73.1 ± 24.4	<0.001
IL-6 (pg/mL)	65.3 ± 18.8	50.9 ± 16.2	<0.001
Ferritin (µg/L)	589 ± 161	475 ± 139	0.001
NLR	7.9 ± 2.7	6.1 ± 2.1	<0.001
Albumin (g/L)	29.9 ± 5.3	33.6 ± 5.5	<0.001
Platelets (×10^9^/L)	198 ± 55	225 ± 60	0.002
CAR	3.10 ± 0.85	2.17 ± 0.72	<0.001
PNI	33.6 ± 4.6	38.2 ± 5.1	<0.001
SII (×10^3^)	1362 ± 450	1087 ± 372	<0.001

CRP—C-Reactive Protein; IL-6—Interleukin-6; NLR—Neutrophil-to-Lymphocyte Ratio; CAR—CRP (mg/L)/Albumin (g/L); PNI—(10 × Albumin [g/L]) + (0.005 × Lymphocyte count [×10^9^/L]); SII—Systemic Immune-Inflammation Index.

**Table 3:** Severity scores (APACHE II, CURB-65, NEWS) at admission.

Variables	COVID and *C. diff* (n = 98)	COVID-Only (n = 142)	*p*-Value
APACHE II	17.1 ± 5.2	13.9 ± 4.8	<0.001
CURB-65	2.6 ± 1.0	2.1 ± 0.9	0.001
NEWS	8.4 ± 2.5	6.9 ± 2.3	<0.001

APACHE II—Acute Physiology and Chronic Health Evaluation II; CURB-65—Confusion, Urea, Respiratory rate, Blood pressure, age ≥ 65; NEWS—National Early Warning Score.

**Table 4:** Subgroup analyses: elderly (≥65 years), diabetes, and cancer.

Subgroup/Outcome	COVID and *C. diff* (n = 98)	COVID-Only (n = 142)	*p*-Value
Elderly (≥65 years) (%)	67.3% (66)	55.6% (79)	0.06
ICU in Elderly (%)	42.4% (28/66)	21.5% (17/79)	0.003
Diabetes (%)	52.0% (51)	40.1% (57)	0.08
ICU in Diabetics (%)	45.1% (23/51)	24.6% (14/57)	0.01
Cancer (%)	17.3% (17)	10.6% (15)	0.14
Mortality in Cancer (%)	41.2% (7/17)	20.0% (3/15)	0.09

ICU—Intensive Care Unit.

**Table 5:** Correlations of inflammatory markers and severity scores with outcomes.

Variable	ICU Admission (r/*p*-Value)	Mechanical Vent (r/*p*-Value)	Mortality (r/*p*-Value)
CRP	0.42/<0.001	0.40/<0.001	0.36/<0.001
IL-6	0.45/<0.001	0.41/<0.001	0.38/<0.001
CAR	0.49/<0.001	0.46/<0.001	0.44/<0.001
PNI	−0.40/<0.001	−0.38/<0.001	−0.42/<0.001
SII	0.35/<0.001	0.33/0.001	0.31/0.002
APACHE II	0.51/<0.001	0.48/<0.001	0.46/<0.001
CURB-65	0.39/<0.001	0.37/<0.001	0.35/<0.001
NEWS	0.43/<0.001	0.40/<0.001	0.37/<0.001

CRP—C-Reactive Protein; IL-6—Interleukin-6; CAR—CRP (mg/L)/Albumin (g/L); PNI—(10 × Albumin [g/L]) + (0.005 × Lymphocyte count [×10^9^/L]); SII—Systemic Immune-Inflammation Index; APACHE II—Acute Physiology and Chronic Health Evaluation II; CURB-65—Confusion, Urea, Respiratory rate, Blood pressure, age ≥ 65; NEWS—National Early Warning Score.

**Table 6:** Logistic regression for independent predictors of severe outcomes.

Variable	Odds Ratio	95% CI	*p*-Value
COVID + CDI	2.6	1.50–4.50	<0.001
IL-6 (per 10 pg/mL increase)	1.22	1.10–1.35	0.001
CAR (per 0.5 increase)	1.38	1.18–1.60	<0.001
PNI (per 1 point decrease)	1.17	1.07–1.28	0.001
Age (per year increase)	1.01	0.99–1.03	0.3
Diabetes Mellitus	1.35	0.88–2.05	0.15
APACHE II (per 1 point)	1.15	1.07–1.24	<0.001

ICU—Intensive Care Unit; CI—Confidence Interval; Severe outcomes defined as ICU admission or mechanical ventilation; CRP—C-Reactive Protein; IL-6—Interleukin-6; CAR—CRP (mg/L)/Albumin (g/L); PNI—(10 × Albumin [g/L]) + (0.005 × Lymphocyte count [×10^9^/L]); SII—Systemic Immune-Inflammation Index; APACHE II—Acute Physiology and Chronic Health Evaluation II; CURB-65—Confusion, Urea, Respiratory rate, Blood pressure, age ≥ 65; NEWS—National Early Warning Score.

**Table 7:** Changes in inflammatory markers and severity scores in surviving vs. non-surviving patients with *C. diff*.

Variable	Survivors (n = 76)	Non-Survivors (n = 22)	*p*-Value
CRP (mg/L)	89.4 ± 25.1	101.2 ± 29.5	0.04
IL-6 (pg/mL)	63.5 ± 17.9	73.3 ± 20.3	0.02
CAR	3.02 ± 0.83	3.33 ± 0.88	0.05
PNI	34.2 ± 4.5	31.9 ± 4.3	0.01
APACHE II	16.4 ± 5.0	19.5 ± 5.3	0.006
CURB-65	2.5 ± 1.0	2.9 ± 1.1	0.11
NEWS	8.1 ± 2.4	9.2 ± 2.6	0.07

CRP—C-Reactive Protein; IL-6—Interleukin-6; CAR—CRP (mg/L)/Albumin (g/L); PNI—(10 × Albumin [g/L]) + (0.005 × Lymphocyte count [×10^9^/L]); APACHE II—Acute Physiology and Chronic Health Evaluation II; CURB-65—Confusion, Urea, Respiratory rate, Blood pressure, age ≥ 65; NEWS—National Early Warning Score.

## Data Availability

The data presented in this study are available on request from the corresponding author.
